# PERSONAL SELF-MONITORING DEVICES TO IMPROVE SLEEP AMONG OLDER PEOPLE: A FEASIBILITY STUDY

**DOI:** 10.1093/geroni/igz038.3348

**Published:** 2019-11-08

**Authors:** Raeann G LeBlanc, Maral Torossian, Paige Czarnecki, Rebecca Spencer, Cynthia Jacelon, Jenna Marquard

**Affiliations:** 1 University of Massachusetts Amherst, Amherst, Massachusetts, United States; 2 University of Massachusetts, Amherst, Massachusetts, United States

## Abstract

Chronic sleep disturbances reduce physical and mental health and affect over 8 million people age 65 years and older in the United States. There is evidence that use of a wearable Personal Self-Monitoring Device (PSMD) may improve sleep self-management in young adult populations. Feasibility of PSMD use for older individuals has not been explored and was the goal of this study. Persons age 65 years and over with self-reported sleep disturbances were recruited in a local community and were asked to wear a commercial PSMD for a 4-week period. To assess whether such an intervention may be feasible, outcomes included consent rate, study completion rate, data download interpretation, identification of a sleep self-management goal, improved knowledge about sleep, and improved sleep. Twenty-six persons (12 males and 14 females) were recruited over 3 months, out of a total of 33 expressing interest. Mean age=72, SD=4.99. Ninety-two percent of participants completed the study and reported improved awareness of sleep patterns and identified a sleep goal. Total sleep time was M=7 hours 14 minutes, SD=40 minutes; total restful sleep time was M=4 hours 33 minutes, SD=1 hour 22 minutes. In conclusion, sleep self-management with the use of a PSMD is feasible and of interest among persons in the young-old age category (65-74 years). There is potential for the use of PSMD among older people with the goal of improved sleep self-management. Future studies for sleep health self-management and interventions using personal sleep monitoring are recommended.

